# Preparation and Thermoelectric Characteristics of ITO/PtRh:PtRh Thin Film Thermocouple

**DOI:** 10.1186/s11671-017-2387-z

**Published:** 2017-12-15

**Authors:** Xiaohui Zhao, Hongmin Wang, Zixiang Zhao, Wanli Zhang, Hongchuan Jiang

**Affiliations:** State Key Laboratory of Electronic Thin Films and Integrated Devices, University of Electronic Science and Technology of China, No.4, Section 2, North Jianshe Road, Chengdu, 610054 China

**Keywords:** ITO/PtRh composite film, Thin film thermocouples, Seebeck coefficients, High-temperature stability

## Abstract

Thin film thermocouples (TFTCs) can provide more precise in situ temperature measurement for aerospace propulsion systems without disturbance of gas flow and surface temperature distribution of the hot components. ITO**/**PtRh:PtRh TFTC with multilayer structure was deposited on alumina ceramic substrate by magnetron sputtering. After annealing, the TFTC was statically calibrated for multiple cycles with temperature up to 1000 °C. The TFTC with excellent stability and repeatability was realized for the negligible variation of EMF in different calibration cycles. It is believed that owing to oxygen diffusion barriers by the oxidation of top PtRh layer and Schottky barriers formed at the grain boundaries of ITO, the variation of the carrier concentration of ITO film is minimized. Meanwhile, the life time of TFTC is more than 30 h in harsh environment. This makes ITO/PtRh:PtRh TFTC a promising candidate for precise surface temperature measurement of hot components of aeroengines.

## Background

121The precise temperature measurement is crucial for aeroengines in order to validate the effectiveness of modeling and simulation of the thermo-mechanical behavior of hot section components and monitor the operating conditions and perform diagnostics [1–3]. Compared with conventional wire thermocouples, infrared photography, or thermal spray instrumentation, thin film thermocouples (TFTCs) could provide precise temperature measurement with fast response, minimal perturbation of the gas flow, and negligible influence on the surface temperature distribution of the measured components [4, 5].

Different materials systems were used to fabricate thin film thermocouples for high-temperature application, such as Pt-PtRh and In_2_O_3_-ITO [6–9]. However, thin film form of these materials is susceptible in stability and repeatability issues, especially at high-temperature range in which aeroengines are generally operated. For instance, selective oxidation of rhodium between 800 and 1000 °C results in the drift and degradation of the Pt-PtRh TFTCs [10, 11]. As for ITO-based TFTCs, although In_2_O_3_-based oxides have the characteristics of higher temperature endurance, the unbalanced compensation of oxygen vacancies would lead to the drift of thermoelectric output and even device failure during high-temperature cycling [12, 13]. Several approaches have been tried to improve the thermoelectric properties of In_2_O_3_-based oxides, such as high-temperature annealing and nitrogen doping [14–16]. The high-temperature stability of ITO-based TFTCs is improved; nevertheless, the thermoelectric output of TFTCs is gradually reduced due to oxygen diffusion in ITO films. In addition, a nanocomposite film comprised of NiCoCrAlY and aluminum oxide has been fabricated and used as the thermoelement for TFTCs [8]. However, the thermoelement with semiconductor/metal multilayer structure has not been reported.

In this work, ITO/PtRh composite film with multilayer structure, which was first introduced as a thermoelement, was prepared by magnetron sputtering and post annealed. The microstructure and resistivity of the films were investigated. Then, ITO/PtRh:PtRh TFTC was fabricated and its thermoelectric response and high-temperature stability were characterized and discussed.

## Methods

### Sample Preparation

ITO thin film and ITO/PtRh composite film were deposited on alumina substrates and Si (100) substrates by magnetron sputtering using high purity ITO (In_2_O_3_:SnO_2_ = 90:10, Ф100 mm, 99.99 wt%) ceramic target and high purity Pt-13%Rh (Ф100 mm, 99.99 wt%) alloy target at room temperature. Table 1 shows the sputtering parameters of ITO and Pt-13%Rh thin films. The background pressure was 7 × 10^−4^ Pa, and the distance between target and substrate was fixed at 110 mm. All substrates were cleaned with acetone, ethanol, and deionized water in sequence before thin film deposition. Specially, ITO and PtRh thin films were deposited alternatively to form ITO/PtRh composite film. The thickness of ITO thin film was nearly four times of Pt-13%Rh thin film and the total thickness of the composite film was approximately 1 μm. In order to improve high-temperature stability of ITO/PtRh composite films, post anneal was performed at 1000 °C for 5 h in nitrogen, followed by annealing at 1000 °C for 2 h in the air (named as N_2_-Air) [15].

**Table 1:** Sputtering parameters of ITO and Pt-13%Rh thin films

Sputtering parameters	Pressure (Pa)	Sputtering	Power (W)	Sputtering media	Temperature
ITO	0.4	RF	150	Ar/O_2_ = 40:2	RT
Pt-13%Rh	0.4	DC	120	Ar	RT

**Fig. 1:**
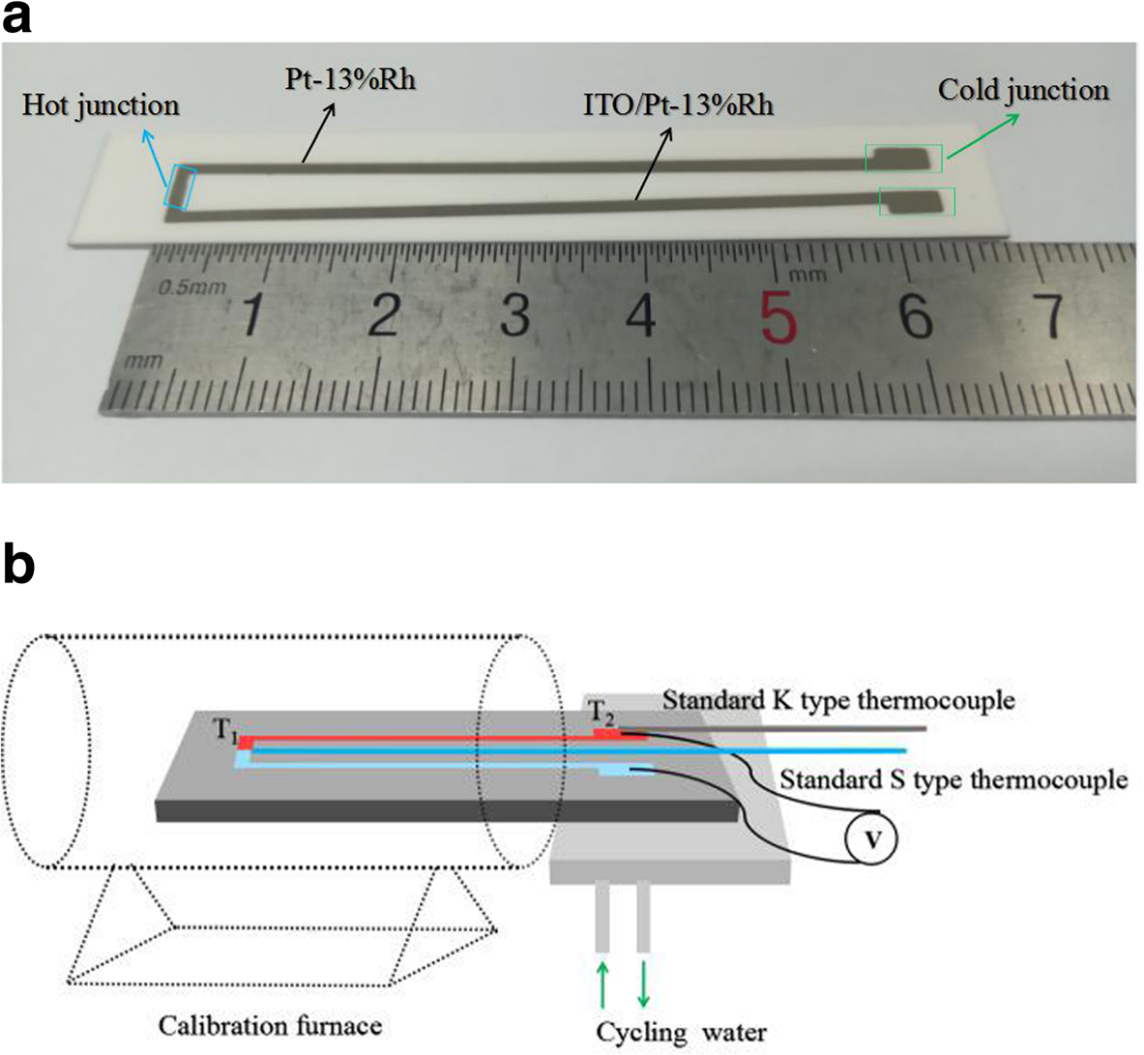
The image of the prepared TFTC (**a**) and the schematic of the calibration system (**b**). **a** The photograph of the ITO/PtRh:PtRh TFTC. It was deposited on alumina substrate (75 mm × 12 mm × 0.5 mm) by magnetron sputtering. Each leg of TFTC is 63 mm in length and 1 mm in width. And the thickness of TFTC is approximately 1 μm. **b** The schematic of the calibration system. The cycling water was used to enlarge temperature gradient between the hot junction and the cold junction. The temperature of hot junction, T_1_, and the temperature of cold junction, T_2_, were measured with standard S and K type wire thermocouples mounted on the back of the substrates, respectively. The cold junction was prolonged by homogeneous wires for connecting a digital multimeter to measure the electromotive force (EMF)

The ITO/PtRh:PtRh TFTC (63 mm × 1 mm × 1 μm) was deposited on 75 mm × 12 mm × 0.5 mm alumina substrate by magnetron sputtering. The thermocouple electrodes were patterned with stenciled masks, and the thickness was approximately 1 μm, as shown in Fig. 1a. After annealing in N_2_-Air, the TFTC was statically calibrated for multiple thermal cycles in the calibration furnace from 300 to 1000 °C. During the calibration, each calibration temperature was retained for at least 1 h in order to reach thermal equilibrium.

### Characterizations

The microstructure of the ITO thin film was characterized by X-ray diffraction (XRD). The scanning electron microscopy (SEM) was applied to disclose the cross-section of ITO/PtRh composite film. The electrical property of the films was measured with four point probe method.

### Calibrating Method

Figure 1b shows the schematic of the calibration system. The TFTC was statically calibrated in the calibration furnace for multiple cycles. The cycling water can be used to enlarge temperature gradient between the hot junction and the cold junction. The temperature of hot junction, T_1_, and the temperature of cold junction, T_2_, were measured with standard S and K type wire thermocouples mounted on the back of the substrates, respectively. The cold junction was prolonged by homogeneous wires for connecting a digital multimeter to measure the electromotive force (EMF).

## Results and Discussion

### Microstructure and Resistivity of ITO and ITO/PtRh Composite Films

**Fig. 2:**
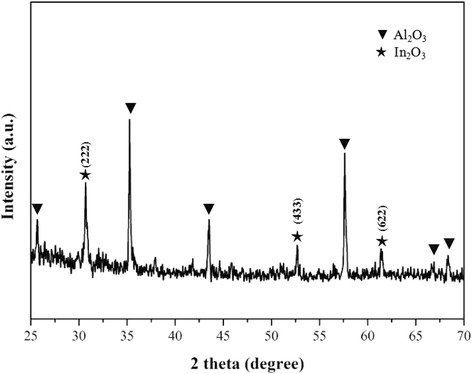
X-ray diffraction patterns of ITO thin film annealed in N_2_-Air. Apart from the peaks of alumina substrate, the diffraction peaks of polycrystalline cubic bixbyite In_2_O_3_ phase have been found without preferred orientation. No diffraction peaks of Sn and corresponding oxide/nitride were observed in the XRD patterns

The XRD patterns of ITO thin film annealed in N_2_-Air are presented in Fig. 2. Apart from the peaks of alumina substrate, the diffraction peaks of polycrystalline cubic bixbyite In_2_O_3_ phase have been found without preferred orientation. No diffraction peaks of Sn and corresponding oxide/nitride were observed in the XRD patterns, confirming that Tin ions were substitutionally doped into the indium oxide lattice and the complete solid solution of In_2_O_3_ and SnO_2_ was formed [17].

**Fig. 3:**
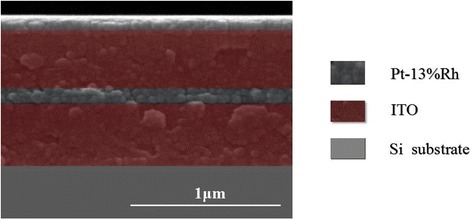
The cross-section SEM image of ITO/PtRh composite film. The cross-section image of ITO/PtRh composite film deposited on Si (100) substrate was obtained with SEM. ITO and Pt-13%Rh films were deposited alternatively to form ITO/PtRh composite film. The total thickness of the composite film was approximately 1 μm and the thickness of single ITO layer was roughly 400 nm, which was four times than that of the thickness of Pt-13%Rh layer

The cross-section image of ITO/PtRh composite film deposited on Si (100) substrate was presented on Fig. 3. ITO and Pt-13%Rh films were deposited alternatively to form ITO/PtRh composite film. The total thickness of the composite film was approximately 1 μm, and the thickness of single ITO layer was roughly 400 nm, which was four times than that of the thickness of Pt-13%Rh layer.

The resistivity of the films was measured by four-point probe method, as shown in Table 2. The resistivity of the as-deposited ITO/PtRh composite film was an order of magnitude smaller than those of the as-deposited ITO film, due to the introduction of PtRh. After annealing in N_2_-Air, the resistivity of the ITO film decreased slightly from 8.52 × 10^−2^ Ω cm to 7.55 × 10^−2^ Ω cm. And this could be contributed to the densification of the film and reduction of defects after annealing. On the contrary, the resistivity of ITO/PtRh composite film increased from 1.68 × 10^−3^ Ω cm to 7.61 × 10^−3^ Ω cm after annealing, which was mainly related to the selective oxidation of Rhodium at the surface of PtRh film during the annealing process [18].

**Table 2:** The resistivity of ITO and ITO/PtRh composite films

Annealing process	As-deposited	N_2_-Air
ITO thin film	8.52 × 10^−2^ Ω cm	7.55 × 10^−2^ Ω cm
ITO/PtRh composite film	1.68 × 10^−3^ Ω cm	7.61 × 10^−3^ Ω cm

### Thermoelectric Properties of ITO/PtRh:PtRh Thin Film Thermocouples

**Fig. 4:**
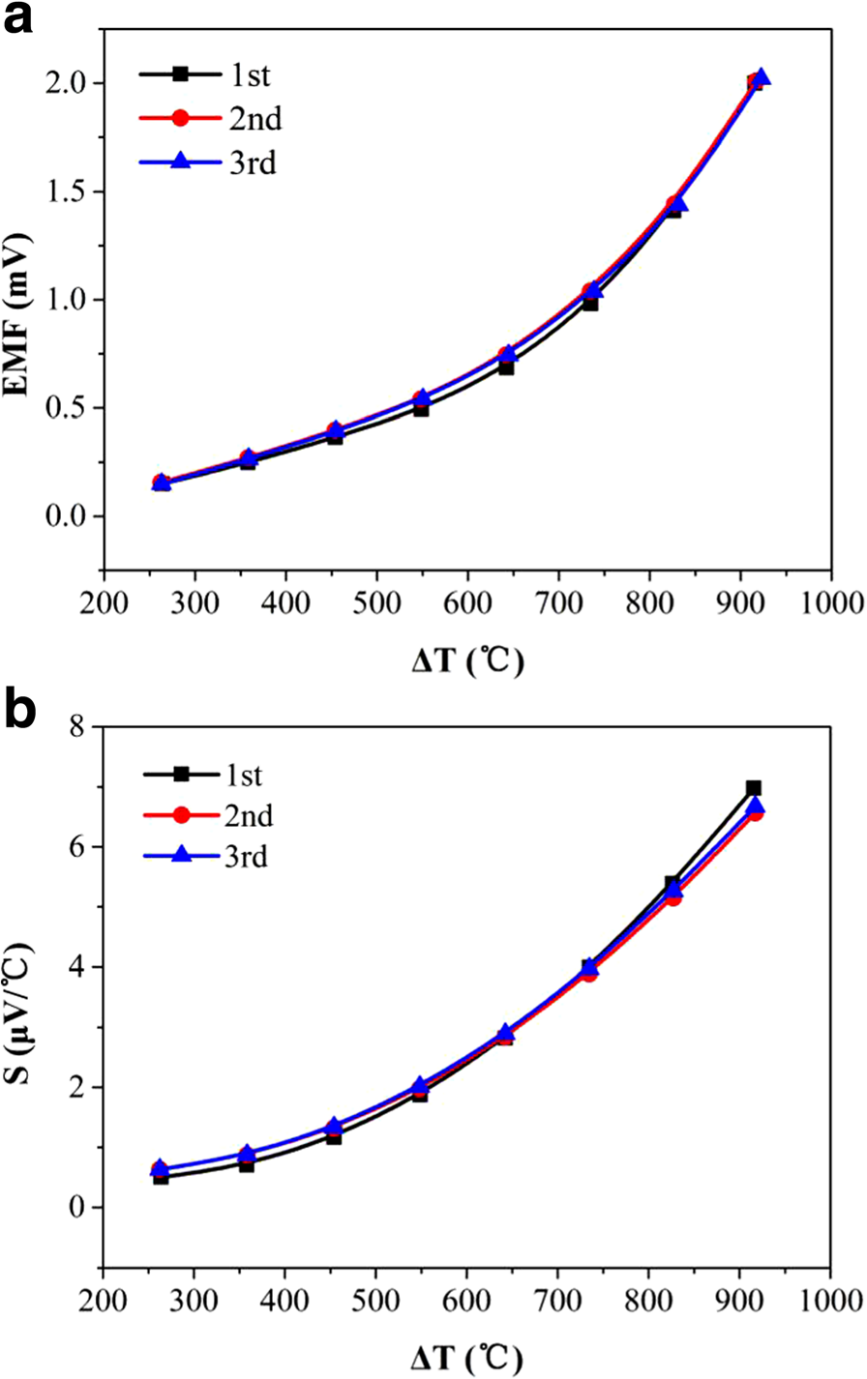
The electromotive force (**a**) and Seebeck coefficients (**b**) of ITO/PtRh:PtRh TFTC. The variation of the electromotive force (EMF) of ITO/PtRh: PtRh TFTC with temperature was shown in **a**. The EMF increased nonlinearly with increasing temperature difference between the hot junction and the cold junction. Negligible variation of EMF in different calibration cycles has been observed, indicating excellent stability and repeatability of TFTC with temperature up to 1000 °C. The Seebeck coefficients of the TFTC with different temperature gradient between hot and cold junction is shown in **b**, which also increased rapidly with increasing temperature gradient. We believe this is mainly induced by the variation of Seebeck coefficient of ITO with increasing temperature. As degenerate semiconductor material, the Seebeck coefficient varies with the degeneration level. The degeneration level would gradually decrease with increasing temperature until the intrinsic excitation occurred. As a result, the absolute value of the Seebeck coefficient of ITO increased significantly with increasing temperature

The static calibration results were shown in Fig. 4. The electromotive force (EMF) of ITO/PtRh: PtRh TFTC increased nonlinearly with increasing temperature difference between the hot junction and the cold junction, as shown in Fig. 4a. Negligible variation of EMF in different calibration cycles has been observed, indicating excellent stability and repeatability of TFTC with temperature up to 1000 °C.

The Seebeck coefficient (*S*) is defined as the slope of the EMF curves at certain temperature. The variation of the Seebeck coefficients of TFTCs as a function of temperature difference is shown in Fig. 4b. Apparently, the Seebeck coefficients of the TFTC also increased rapidly with increasing temperature difference. We believe this is mainly induced by the variation of Seebeck coefficient of ITO. As degenerate semiconductor material, the Seebeck coefficient varies with the degeneration level. The degeneration level would gradually decreased with increasing temperature until the intrinsic excitation occurred. As a result, the absolute value of the Seebeck coefficient of ITO increased significantly with increasing temperature [19, 20].

Compared with traditional S-type or R-type thermocouples, the Seebeck coefficient of the TFTC was smaller. We believe that it can be ascribed to Schottky barriers between ITO and PtRh which may change transport characteristics of carriers in ITO/PtRh composite film [21, 22]. The Seebeck coefficient of oxide semiconductor is highly dependent on the carrier concentration [23]. As a degenerate semiconductor, the Seebeck coefficient of ITO can be described by Eq. (1):

1 $$ S\left({N}_D\right)=-{\left(\frac{\pi }{3{N}_D}\right)}^{\raisebox{1ex}{$2$}\!\left/ \!\raisebox{-1ex}{$3$}\right.}\frac{8{k}^2{m}^{\ast }T}{3e{\mathrm{\hslash}}^2}\left(A+\frac{3}{2}\right) $$

where *S*(*N*_*D*_) is the Seebeck coefficient, *k* is the Boltzmann constant, *T* is the absolute temperature, *N*_*D*_ is the carrier concentration, *e* is the electron elementary charge, *m* ^∗^is the effective mass, *ħ* is the reduced Planck constant, and *A* is the transport constant [3, 7]. Thus, minimization of variations in the carrier concentration is an essential prerequisite for TFTCs with excellent stability and repeatability. Apart from the substitution of Tin ions, carriers in ITO film are ordinarily attributed to oxygen vacancies, as shown in Eq. (2). Oxygen vacancies become the major factor affecting carrier concentration in fixed ingredient ITO film.

2 $$ {O}_O^x\iff {V}_O^{\bullet \bullet }+2{e}^{\hbox{'}}+\frac{1}{2}{O}_2\left(\mathrm{g}\right) $$

The selective oxidation of rhodium at the surface of top PtRh layer forms oxygen diffusion barriers, which isolates ITO layer from the external oxygen environment. Meanwhile, Platinum and Rhodium atoms would diffuse into ITO film at high temperature and segregate at the grain boundary of ITO film. As a consequence, the Schottky barriers could form at the grain boundaries of ITO. The Schottky barriers may constrain the local concentration of oxygen vacancies in ITO film. As a result, the variation of the carrier concentration of ITO film is minimized. All of these factors lead to excellent high-temperature stability and repeatability of the thermoelectric response of the TFTC.

The relationship between thermoelectric response and temperature difference could be described according to the following third order polynomial expression:

3 $$ E\left(\Delta T\right)=A{\left(\Delta T\right)}^3+B{\left(\Delta T\right)}^2+C\left(\Delta T\right)+D $$

where Δ*T* is the applied temperature difference between the hot junction and the cold junction of TFTCs. *A*, *B*, *C*, and *D* are polynomial constants. *D* is sedulously set to zero to meet the boundary condition (E (Δ*T*) = 0, if Δ*T* = 0).

The fitting results of TFTC are shown in Table 3. The coefficients of different calibration cycle are close, indicating excellent stability and repeatability of the TFTC. The average Seebeck coefficients of three calibration cycles were 2.19 μV/°C. We believe this is related to the Schottky barrier formation at grain boundaries. The Schottky barrier would not only stabilize the oxygen vacancies in ITO, but also intensify the grain boundary scattering of the charge carriers of ITO, which plays a major role in ITO films especially at high-temperature range [24]. As a result, the Seebeck coefficients of TFTC decreased. Despite of this, the TFTC remained in good condition after multiple calibration cycles with temperature up to 1000 °C, meaning that the life time of ITO/PtRh:PtRh TFTC is more than 30 h in harsh environment. This makes ITO/PtRh:PtRh TFTC a promising candidate for precise surface temperature measurement of hot components of aeroengines.

**Table 3:** Polynomial fitting results of electromotive force of ITO/PtRh:PtRh TFTC

Coefficients of polynomial *E(ΔT) = A(ΔT)* [3] *+ B(ΔT)* [2] *+ C(*Δ*T) + D*						
Calibration number	*A* (mV/°C^3^)	*B* (mV/°C^2^)	*C* (mV/°C)	*D* (mV)	Fitting coefficient *R* [2]	Average Seebeck coefficient (μV/°C)
1st	4.65 × 10^−9^	− 3.26 × 10^−6^	1.25 × 10^−3^	0	0.999	2.19
2nd	3.93 × 10^−9^	− 2.44 × 10^−6^	1.10 × 10^−3^	0	0.999	2.19
3rd	3.96 × 10^−9^	− 2.43 × 10^−6^	1.09 × 10^−3^	0	0.999	2.19

## Conclusions

In summary, ITO thin film and ITO/PtRh composite film were deposited on alumina substrate by magnetron sputtering at room temperature and annealed. The resistivity of the ITO film decreased slightly after annealing, while the resistivity of ITO/PtRh composite film increased significantly to the selective oxidation of Rhodium at the surface of PtRh film. The ITO/PtRh:PtRh TFTC with multilayer structure was fabricated and statically calibrated from 300 to 1000 °C. Due to oxygen diffusion barriers by the oxidation of top PtRh layer and Schottky barriers formed at the grain boundaries of ITO, the variation of the carrier concentration of ITO film is minimized, which leads to excellent high-temperature stability and repeatability of the TFTC. The average Seebeck coefficients in 3 cycles calibration were 2.19 μV/°C and the life time of the TFTC is more than 30 h in harsh environment. It is worthwhile to note that apart from high-temperature annealing and nitrogen doping, a new method is available to improve the stability of thermoelectric properties of ITO film, especially at high-temperature range in which aeroengines are generally operated.

## Abbreviations

EMF: Electromotive force
S: Seebeck coefficient
SEM: Scanning electron microscopy
TFTCs: Thin film thermocouples
XRD: X-ray diffraction
